# Identification of Ovarian Circular RNAs and Differential Expression Analysis between MeiShan and Large White Pigs

**DOI:** 10.3390/ani10071114

**Published:** 2020-06-29

**Authors:** Guoming Liang, Junyu Yan, Jin Guo, Zhonglin Tang

**Affiliations:** 1 State Key Laboratory of Animal Nutrition, Institute of Animal Science, Chinese Academy of Agricultural Sciences, Beijing 100193, China; liangguoming@caas.cn (G.L.); 82101181031@caas.cn (J.Y.); 82101175134@caas.cn (J.G.); 2 Genome Analysis Laboratory of the Ministry of Agriculture, Agricultural Genome Institute at Shenzhen, Chinese Academy of Agricultural Sciences, Shenzhen 518120, China; 3 Guangdong Laboratory of Lingnan Modern Agriculture, Shenzhen 518120, China

**Keywords:** pig, circRNA, ovary, differential expression, fecundity

## Abstract

**Simple Summary:**

Chinese MeiShan pigs make up one of the oldest Chinese pig breeds. Compared with Large White pigs, MeiShans are hyper-productive and prolific, however, the expression and function of circular RNAs (circRNAs) in these two breeds of pig are not yet fully understood. We performed RNA-seq analysis of porcine ovaries from three MeiShan sows and three Large White sows, identifying 85 differentially expressed circRNAs (*p* < 0.05). In MeiShan pigs, 37 circRNAs were significantly up-regulated and 48 circRNAs were down-regulated. We verified that circular RNA scinderin (circSCIN) can target and sponge miR-133 and miR-148a/b, two miRNAs that affect estrogen secretion. This study highlights the circRNA profiling of ovaries and helps investigators to understand the differences in reproductive efficiency between MeiShan and Large White pigs.

**Abstract:**

MeiShan and Large White pigs differ in their female fecundity. However, the mechanisms behind the gene expression and regulation that cause these differences remain unclear. In this study, we profiled circRNAs and identified 5,879 circRNAs from the ovaries of MeiShan and Large White pigs. Eighty-five circRNAs were differentially expressed between the two pig breeds. Of these, 37 were up-regulated and 48 were down-regulated in MeiShan pigs. Gene ontology enrichment analysis suggested that the differentially expressed circRNA were involved in the hormone-mediated signaling pathway. We verified that *circSCIN* and its parent gene, scinderin (*SCIN*), were differentially expressed by reverse transcription and quantitative PCR (RT-qPCR). Luciferase assays demonstrated that *circSCIN* can target and sponge miR-133 and miR-148a/b. The identification of differentially expressed circRNAs (DECs) and their regulatory functions increased our understanding of the differences in reproductive efficiency between MeiShan and Large White pigs.

## 1. Introduction

Sow fecundity is an important trait in the pig farming industry and has a direct economic impact for farmers. MeiShan pigs are well known for their high fecundity and consistently large litter sizes, compared to other pig breeds [1]. Several genes, including *ESR* [2], *IGF1R* [3], and *BMPR1B* [4], are expressed in the ovary and are reported to cause genetic differences in fecundity between pig breeds. However, the transcriptome differences among pig breeds are not well characterized. Factors such as ovarian hormone levels, follicular development, mating time, and ovulation rate reportedly affect sow fecundity during the estrous stage [5,6]. In the follicular phase, ovarian follicular growth and differentiation are affected by the transcriptional regulation of genes due to changes in hormone binding [7] or steroidogenic mediator genes (e.g., *CYP11A1* and *STAR*) [8]. During pregnancy, the ovary continues to synthesize hormones essential for the maintenance of conceptus. One study indicated that differentially expressed genes (DEGs) between MeiShan and Iberian pigs were colocalized with a QTL for litter size, suggesting that DEG regulation was associated with litter size [9]. Moreover, some DEGs are also correlated with differences in fetus weight and length across different pig breeds [10]. There is currently insufficient evidence to suggest that maternal hormone levels directly affect fetus growth; however, this hormone may increase fetal growth by enhancing the expression of placental nutrient transporter proteins [11]. As such, genes related to hormone secretion or energy metabolism may be differentially expressed between pig breeds with different fecundities. 

CircRNA is a type of nonlinear and noncoding RNA with a covalently closed loop structure formed by the ligation of a downstream 5′ splice donor site with an upstream 3′ acceptor site [12]. As RNA deep sequencing technology and bioinformatic technologies have progressed, several circRNAs have been identified in a variety of eukaryotic organisms [13,14,15]. In pigs, Liang et al. identified thousands of circRNAs in the ovary of a mini-pig and reported that some circRNAs were ovary-specific [13], but no further studies were performed to determine the regulatory function of these circRNAs in porcine ovaries. In addition to circRNAs, various studies have reported that noncoding RNAs, such as miRNAs [16,17,18] and lncRNAs [19], are also differentially expressed between pig breeds. CircRNAs contain miRNA binding sites and may function as miRNA sponges to indirectly regulate the target gene [20]. The regulation of circRNA as miRNA sponges has been widely demonstrated in cancer studies [21], but in pigs, the role of circRNAs requires further investigation. 

In this study, we used RNA sequencing (RNA-seq) to investigate the circRNA expression profiles of the ovarian tissues in MeiShan and Large White pigs 49 days after coitus. Our goal was to identify and characterize the differentially expressed circRNAs in porcine ovaries and to better understand their regulatory function in sow reproduction. 

## 2. Materials and Methods

### 2.1. Animals

This study was authorized by the Animal Welfare and Ethical Committee of the Institute of Animal Sciences at the Chinese Academy of Agricultural Sciences (IAS-CAAS, Beijing, China). All methods and procedures were performed on the experimental animals in accordance with the relevant guidelines (document approval number IAS20160605). Three Large White and three MeiShan sows were raised in the same environment and were all in the same parity pregnant. Six sows were slaughter at 49 days post coitus. We checked that all the ovaries were normal, and was immediately frozen in liquid nitrogen and then was transported to our laboratory within 3 hours and stored at −80 °C until use.

### 2.2. RNA Sequencing

RNA-seq analysis was performed on the ovaries from three MeiShan sows and three Large White sows. Total RNA was extracted from the ovary samples using TRIzol reagent (Invitrogen) according to the manufacturer’s instructions. Genomic DNA was removed using DNaseI (Qiagen). RNA quantity and quality were assessed using an Agilent 2100 Bioanalyzer (Agilent Technologies). Ribosomal RNA was depleted using a Ribo-Zero Magnetic Kit (Epicentre). Six cDNA libraries were constructed from ovarian RNA and sequenced on an Illumina HiSeq 2500 platform to generate 125-bp paired-end reads. The sequences were submitted to the Gene Expression Omnibus (GEO) database under the accession code GSE73593.

### 2.3. Sequence Mapping and Identification of Differentially Expressed Genes

We used the hisat2 package [22] to map raw reads to the *Sus scrofa* reference genome (version 11.1). Expression levels of mRNAs were quantified as Transcripts Per Kilobase Million (TPM) using the StringTie package [22]. The read counts for the genes were collected using HTSeq [23] according to the *Sus scrofa* GTF file (version 11.1). We used the edgeR software package [24] to identify DEGs. All P-values were adjusted for false discovery rate (FDR), since multiple testing procedures were used to control for type I errors. The level of statistical significance for DEGs was set with cutoffs of |log_10_(FC)|≥1, *p* ≤ 0.05, and FDR ≤ 0.05, where “FC” indicates fold-change in expression for circRNA.

### 2.4. Identification and Verification of circRNAs

The pipeline and software used to identify circRNAs were described in our previous study [13]. A putative circRNA was supported by at least two raw reads across its junction region. Differentially expressed circRNAs were identified by the edgeR software package [24], with cutoffs of |log_10_(FC)| ≥ 1, *p* ≤ 0.05, and FDR ≤ 0.5, where “FC” indicates fold-change in expression for the gene.

The primers SCIN-F (5′-TCACAAATGACTTGAGCGCC-3′) and SCIN-R (5′-GCTTAGGGGAACTTCCGTGG-3’) were used for *SCIN* amplification. The divergence primers circSCIN-F (5′-ACTGGCATTCGGGACAATGA-3′) and circSCIN-R (5′-ACTGGTTTGCCGCCCAAATA-3′) were used for *circSCIN* amplification. 

### 2.5. Identification of Potential Target miRNAs

We used miRanda [25], microTar [26], and RNAhybird [27] to predict miRNA targets for the identified circRNAs. Targeted miRNAs were supported by the predictions of at least two programs.

### 2.6. Gene Ontology (GO) Enrichment Analysis

We used DAVID web tools to identify enriched biological terms [28]. The DEGs were mapped to GO terms in the GO database using a hypergeometric test. The threshold was set as *p* ≤ 0.05 and was corrected using the Benjamini-Hochberg (BH) procedure. 

### 2.7. Transfections and Luciferase Reporter Assays

Synthetic miRNA mimics were purchased from RiboBio Company (Guangzhou, Guangdong, China). The miRNA mimics were transfected into HEK 293T cells using the Lipofectamine 2000 transfection system (Invitrogen). The transfection of plasmids into HEK 293T cells was performed with the Lipofectamine 2000 transfection reagent according to the manufacturer’s instructions. The wild-type psiCHECK2-circSCIN-WT construct was generated by inserting the circSCIN fragments containing the miRNA binding sequence into the psiCHECK-2 vector (Promega) at the 3′ end of the Renilla luciferase gene. The mutant psiCHECK2-circSCIN-MUT construct was generated by mutating the miRNA-binding sequence to the complementary sequence using overlapping extension PCR. For circSCIN luciferase assays, the HEK 293T cells were transfected with miRNA mimics and either the psiCHECK2-circSCIN-WT or mutated psiCHECK2-circSCIN-Mutreporter plasmid. At 48 h post-transfection, luciferase activity was measured using a dual-luciferase reporter assay system (Promega) according to the manufacturer’s instructions. The relative luciferase activities were calculated by comparing the Firefly/Renilla luciferase activity ratio.

## 3. Results

### 3.1. Identifying circRNA and Differential Expression Analysis

**Figure 1:**
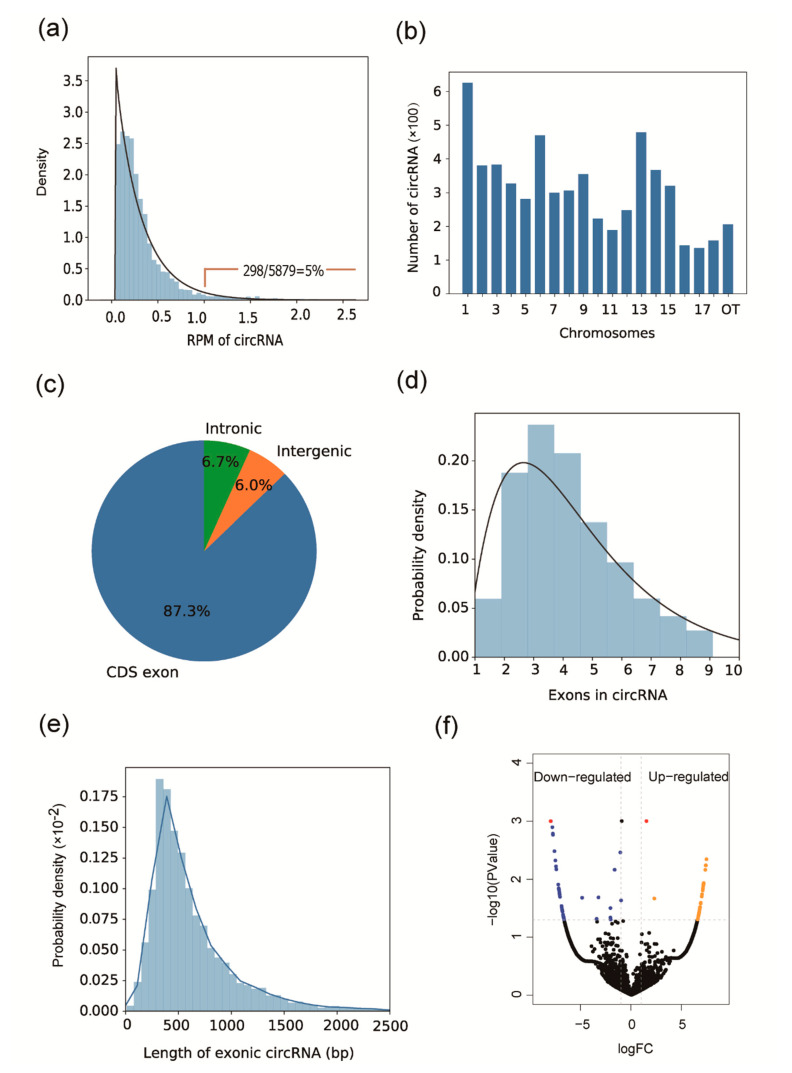
Circular RNAs (CircRNA) characteristics and volcano plot. (**a**) The distribution of Reads Per Million (RPM) for circRNAs. (**b**) The genomic location of circRNA in porcine chromosomes. Others (OT) represent a circRNA that was on scaffold and had not been assigned a chromosome. (**c**) Categories of circRNAs based on their genomic origin. (**d**) Distribution of the exon numbers of circRNAs. (**e**) Distribution of exonic circRNA length. (**f**) Volcano plot of differentially expressed circRNAs (DECs). The blue and orange dots represent down- and up-regulated circRNA, respectively. The red point represents DECs with a straight cutoff of [|log10(FC)| ≥ 1, *p* ≤ 0.05, and FDR ≤ 0.05].

The sequencing of the mRNA libraries generated 250,823,848 raw 125-bp paired-end reads, resulting in 59.80 giga bases of expression data. The reads were mapped to the *Sus scrofa* reference genome (version 11.1). The circRNA identification pipeline used in this study referenced our previous study [13]. We defined circRNAs as having at least two reads across a junction region. In total, 5879 circRNAs were identified and originated from 2454 genes (Table S1 and Table S2). All of these circRNAs were submitted to a pig circRNA database [13]. We used Reads Per Million (RPM) map reads to normalize circRNA abundance. Among the normalized circRNAs, only 298 circRNAs (298/5879 = 5%) had an RPM ≥ 1 in at least one sample (n = 6, Figure 1a), indicating that these circRNA display a low expression abundance. The circRNA genomic location in the chromosomes did not differ from our previous study (Figure 1b). The percentage of exonic circRNA was 87.3%, while percentages of intronic and intergenic region circRNA were 6.7% and 6.0%, respectively (Figure 1c). More than 90% of circRNAs are formed by multiple exons and for exonic circRNAs, and most circularizing events typically encompass fewer than 5 exons (Figure 1d). The distribution of circRNA exonic sequence lengths is similar to that of our previous study, with a median length of 500 nucleotides (Figure 1e). 

Eighty-five circRNAs are differentially expressed between the two pig breeds [|log_10_(FC)| ≥ 1, *p* ≤ 0.05, and FDR ≤ 0.5]. Of these, 37 circRNAs are up-regulated and 48 circRNA are down-regulated in MeiShan pigs. We used a strict cutoff [|log_10_(FC)| ≥ 1, *p* ≤ 0.05, and FDR ≤ 0.05] to filter differentially expressed circRNAs, and identified only three circRNAs that were differentially expressed in MeiShan and Large White pigs (Figure 1f and Table S3). The up-regulated circRNA in MeiShan pigs is circLOC100513601, and is located at chr7:22889305-22956239. Two circRNAs (*circSCIN* and *circTXN2*) are down-regulated in MeiShan pigs, and are located at chr9:82118876-82135701 and chr5:11265443-11268764, respectively. 

### 3.2. Gene Ontology Enrichment Analysis of Differentially Expressed circRNAs

**Figure 2:**
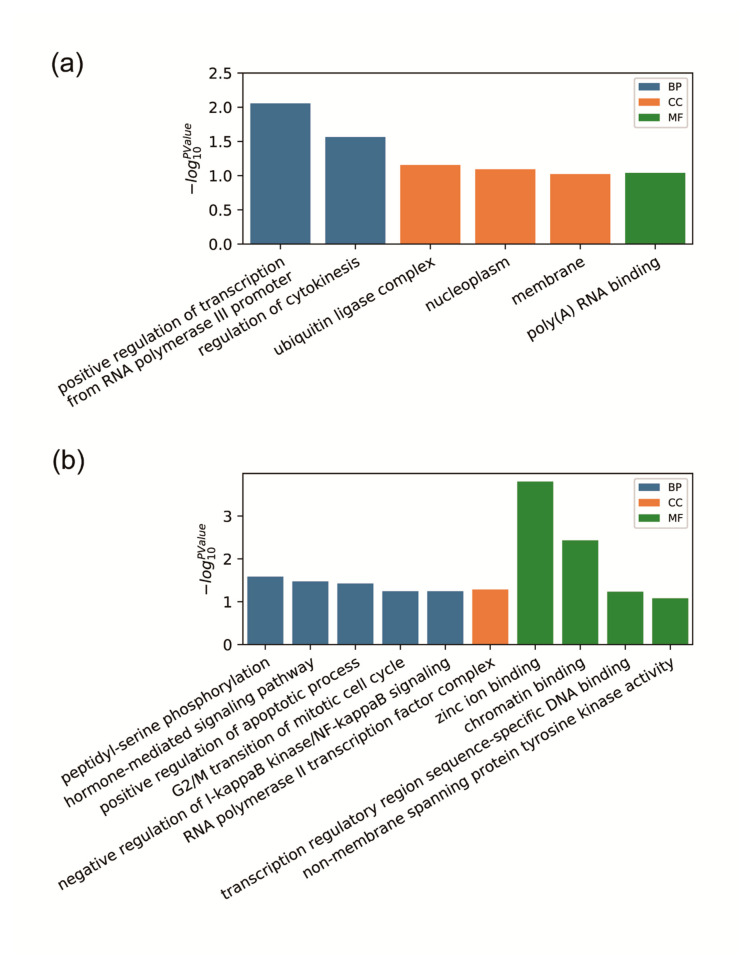
Gene ontology (GO) term enrichment analysis. (**a**) Enrichment GO terms for up-regulated circRNAs. (**b**) Enrichment GO terms for down-regulated circRNAs. BP, biological process, CC, Cellular Component, MF, and molecular function.

To understand differentially expressed circRNAs and their biological functions, we performed GO enrichment analysis for up-regulated and down-regulated circRNA. We also performed a functional annotation to identify high-confidence GO terms, including biological process (BP), cellular component (CC), and molecular function (MF). The up-regulated circRNA was involved in positively regulating the transcriptions from the RNA polymerase III promoter 2, the regulation of cytokinesis, the ubiquitin ligase complex, the nucleoplasm, the membrane, and poly(A) RNA binding (Figure 2a). For down-regulated circRNA, the enriched GO terms were related to peptidyl-serine phosphorylation, positive regulation of the apoptotic process, G2/M transition of the mitotic cell cycle, the RNA polymerase II transcription factor complex, zinc ion binding, chromatin binding, and transcription regulatory region sequence-specific DNA binding (Figure 2b)**.** Most importantly, the GO term of the hormone-mediated signaling pathway was related to hormone secretion in the ovaries, leading us to speculate that these circRNAs were associated with sow fecundity. 

### 3.3. Verifying circSCIN and SCIN Differential Expression

**Figure 3:**
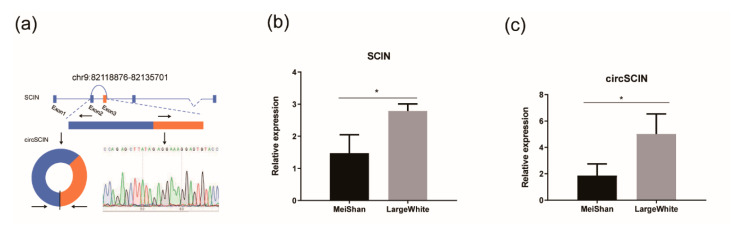
RT-qPCR analysis of the expression levels of circSCIN and its parent gene *SCIN*. (**a**) The genomic location of circSCIN and Sanger sequencing of a PCR product resulting from divergent primers for demonstrating the backsplicing of two exons in *SCIN*. (**b**) The expression of circSCIN was significantly increased in Large White pigs. (**c**) The abundance of *SCIN* was significantly increased in Large White pigs. Data in (**b**) and (**c**) were the means ± s.e.m. * *p* ≤ 0.05 (Student’s *t*-test).

We analyzed the differentially expressed mRNA (Table S4) and found *circSCIN* and its parent gene, *SCIN*, were both differentially expressed between MeiShan and Large White pigs [|log10(FC)| ≥ 1, *p* ≤ 0.05, and FDR ≤ 0.05]. *SCIN* is located on chromosome 9, and *circSCIN* is generated via the ligation of the 3’ donor of the third exon to the upstream 5’ acceptor of the second exon, which forms an 1103-nt nucleotide circRNAs (Figure 3a). We first designed a divergence primer and verified the putative *circSCIN* junction by Sanger sequencing (Figure 3a). Subsequently, we detected *circSCIN* and *SCIN* expression using real-time quantitative PCR and verified that both *circSCIN* and *SCIN* were differentially expressed between MeiShan and Large White pigs (Figure 3b,c). 

### 3.4. CircSCIN binds to miR-133 and miR-148a/b

**Figure 4:**
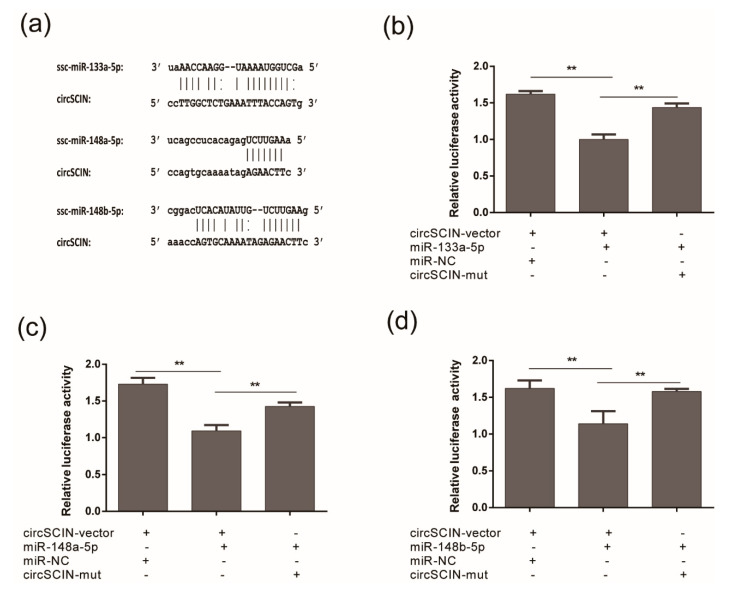
CircSCIN sponged with miR-133a and miR-148a/b. (**a**) The predicted miR-133a-5p, miR-148a-5p, and miR-148b-5p binding sites at three distinct positions in circSCIN. (**b**), (**c**), (**d**) Luciferase reporter assay revealed that miR-133a, miR-148a, and miR-148b were able to reduce the luciferase activity. “+” and “-” in (**b**), (**c**), and (**d**) represented circSCIN-vector, miRNA mimic, miR-NC and circSCIN-mut was transfected into HEK 293T cells or not, respectively. Data in (**b**), (**c**), and (**d**) were the means ± s.e.m for relative luciferase activity. ** *p* ≤ 0.01 (Student’s t-test).

CircRNA contains miRNA binding sites and functions as a sponge. To verify this, we searched 447 miRNA sequences in the miRBase database [29]. Among the target miRNAs, ssc-miR-148a-5p, ssc-miR-148b-5p, ssc-miR-133a-5p, ssc-miR-133b, ssc-miR-218, ssc-miR-218b, and ssc-miR-218-5p were all identified by miRanda, microTar, and RNAhybrid as possible *circSCIN* targets. Of these, *circSCIN* is predicted to contain three binding sites for each miR-133a and miR-148a, and one binding site for miR-148b (Figure 4a).

Next, we used luciferase assays to verify these putative binding sites. We mutated each miRNA target site in a luciferase reporter that included the *circSCIN* sequence in the 3’ UTR, and expressed these alongside a *circSCIN* expression vector. We observed that miR-133a-5p, miR-148a-5p, and miR-148b-5p all significantly inhibited the Rluc (Renilla luciferase) expression of pCK-circSCIN (Figure 4b–d). These results suggest that *circSCIN* could function as a sponge of miR-133a and miR-148a/b.

## 4. Discussion

In this study, we used RNA-seq to investigate differentially expressed circRNAs between the ovaries of MeiShan and Large White pigs. We identified 85 differentially expressed circRNAs. The identification of these DECs indicates that there is a difference in sow fecundity between MeiShan and Large White pigs. Sow fecundity is affected by several limiting factors, including estrus. In this study, GO analyses revealed the enrichment of estrogen response, suggesting that DECs are involved in estrus by indirectly regulating the target gene. A similar study identified several genes related to estrus. Huang et al. investigated gene expression in the porcine endometrium during three gestational stages (gestational days 15, 26, and 50) and identified 228 DEGs between MeiShan and Large White endometria. The expression patterns of these DEGs revealed a divergence in the endometrial environment between the two breeds [30]. Yang et al. compared the gene expression profiles of the proestrus and estrus stages. The authors identified more down-regulated genes in the estrus group when compared to the proestrus group, and demonstrated that the sow estrous cycle affects sow fecundity [31]. These two studies indicate that several genes could affect estrus. For example, inhibin beta A (*INHBA*) can inhibit follicle-stimulating hormone (FSH) secretion and activity in granulosa cells and was found to be associated with litter size [32]. *INHBB* was also identified and is considered a candidate marker gene for infertility in domestic animals [33,34]. These results suggested that estrus regulation drives differences in female fecundity between pig breeds. Thus, many differentially expressed coding and noncoding RNAs are widely expressed in the proestrus or estrus stages and result in differences in litter size and prolificacy between pig breeds [16]. 

SCIN is a Ca^2+^-dependent protein belonging to the gelsolin superfamily. It also controls actin dynamics [35,36]. Sperm capacitation and the acrosome reaction are import processes for mammalian fecundity; only capacitated sperm can undergo the acrosome reaction and penetrate the egg to fertilize it. During this process, globular (G)-actin polymerizes into filamentous (F)-actin, and SCIN is one of the actin-binding proteins that controls this polymerization [37,38]. Thus, SCIN could be involved in regulating porcine fecundity and cause differences in fecundity between MeiShan and Large White sows. 

We demonstrated that *SCIN* can generate a circRNA. Since it is a newly verified circRNA, the function of *circSCIN* regulation was not well characterized. It has been reported that circRNAs function as sponges for targeting miRNAs [39]. Previous studies revealed numerous miRNAs involved in regulating female reproductive hormone signaling during estrus [40,41]. In this study, we verified that *circSCIN* can sponge and bind to miR-133 and miR-148a/b. MiR-133 has been demonstrated to regulate oocyte meiosis [42] and suppress ovarian cancer cell proliferation [43,44]. MiR-133b has been reported to be involved in FSH-induced estrogen secretion [45]. Another study reported that miR-133b can stimulate ovarian estradiol synthesis by targeting and down-regulating *Foxl2* expression in human and mouse granulosa cells [46]. An increasing amount of evidence suggests that miR-148 can regulate ovarian carcinoma [47,48,49]. We speculate that the regulation of *circSCIN* causes differences in reproductive performance between MeiShan and Large White pigs. 

## 5. Conclusions

Our study identified 85 differentially expressed circRNAs between MeiShan and Large White pigs. Of these, 37 were up-regulated and 48 circRNA were down-regulated in MeiShan pigs. The expression of circSCIN and its parent gene, *SCIN*, was verified by RT-PCR. We also found that circSCIN could bind with miR-133 and miR-148a/b. Our results provided differentially expressed circRNAs, and help to understand the differences in reproductive efficiency between MeiShan and Large White pigs.

## Supplementary Materials

The following are available online at https://www.mdpi.com/2076-2615/10/7/1114/s1, Table S1: Summary of RNA sequencing and reads mapping, Table S2: The genomic location and its parent gene for circRNAs, Table S3: Summary of the differential expression analysis for circRNAs, Table S4: Summary of the differential expression analysis for protein-coding genes.
